# Cardiac magnetic resonance imaging in systemic sclerosis: Heart involvement in high-resolution

**DOI:** 10.1515/rir-2024-0011

**Published:** 2024-07-15

**Authors:** Jessica L Fairley, Rachael O’Rourke, Rajesh Puranik, Mandana Nikpour

**Affiliations:** The University of Melbourne, Melbourne, Victoria, Australia; St. Vincent’s Hospital Melbourne, Melbourne, Victoria, Australia; The Prince Charles Hospital, Brisbane, Queensland, Australia; The University of Queensland, Brisbane, Queensland, Australia; The University of Sydney, Sydney, New South Wales, Australia; Royal Prince Alfred Hospital Sydney, New South Wales, Australia

**Keywords:** systemic sclerosis, cardiac magnetic resonance imaging, scleroderma heart involvement

## Abstract

Cardiac magnetic resonance imaging (CMR) is the gold-standard non-invasive method of assessing cardiac structure and function, including tissue characterisation. In systemic sclerosis (SSc), heart involvement (SHI) is a leading cause of mortality yet remains poorly understood. SHI is underestimated by conventional echocardiography, and CMR provides an important opportunity to better identify and quantify subtle myocardial changes including oedema and fibrosis. This review summarises current CMR techniques, the role of CMR in SSc and SHI, and the opportunities to further our understanding of its pathogenesis and management.

## Introduction

Systemic sclerosis (SSc) is an autoimmune disorder characterised by multiorgan involvement, fibrosis and tightening of the skin, as well as fibrosis of internal organs.^[1]^ SSc has the highest case-based mortality of all rheumatic diseases, and significantly reduced life expectancy.^[2]^ SSc Heart Involvement (SHI) is associated with a poor prognosis,^[3,4]^ but remains a poorly understood and under-recognised complication of SSc.

Autopsy studies have shown inflammatory, fibrotic and vascular changes within the myocardium, even among those without cardiac symptoms in life.^[5]^ Myocardial fibrosis can be identified in up to 80% of autopsy studies,^[5]^ suggesting a large ‘subclinical’ burden of SHI. Furthermore, echocardiography alone is inadequate to detect this myocardial fibrosis, with over 90% of patients with normal transthoracic echocardiography (TTE) having a pathological burden of myocardial fibrosis on cardiac magnetic resonance imaging (CMR).^[6]^

This discrepancy highlights the needM for advanced imaging techniques, particularly CMR, to help understand and identify SHI given its prognostic impact. This review provides an overview of CMR techniques, and explores its role in understanding and managing SHI.

## What is CMR?

CMR provides gold-standard non-invasive structural and functional imaging of the myocardium including tissue characterisation. CMR utilises either 1.5 Tesla (T) or 3T scanners with vector (VCG)-gating and dedicated software to provide detailed images of the myocardium.^[7,8]^ Different parameters are assessed utilising different CMR sequences (Table 1).

**Table 1:** Myocardial tissue characterisation using CMR.

Technique	Utility	Pitfalls
LGE	Gadolinium highlights areas of increased extracellular volume Identifies areas of focal myocardial fibrosis, necrosis, oedema or inflammation	More difficult to identify subtle diffuse processes
T1 mapping	Native T1 Abnormalities of both intra- and extra-cellular space (*e.g*., hypertrophy, oedema, fibrosis or infiltrative processes) Doesn’t require gadolinium contrast	Less sensitive to abnormalities of only extracellular space Depends on exact local set-up; values not generalisable Abnormal values have significant overlap with normal patients.
	Postcontrast T1 Allows calculation of volume of extracellular space alone	Requires gadolinium contrast Prone to artefact in setting of arrythmia and breathing movement.
ECV fraction	Based on pre- and post-contrast T1 mapping Quantifies the proportion of myocardium that is extracellular to identify processes that expand the extracellular space Particularly good in detecting diffuse sub-clinical fibrosis and infiltrates	Requires gadolinium contrast Removes issue of overlap with normal patients with high T1 native values.
T2 mapping	More sensitive to oedema than T1 mapping and T2 DIR sequences alone Particularly good at detecting global inflammation which can occur in CTD	Can help overcome false-negative results of LGE and T2 DIR visual assessment in those with myositis

CMR, cardiac magnetic resonance imaging; CTD, connective tissue disease; ECV, extracellular volume; LGE, late gadolinium enhancement.

### Structure and Functional Imaging

The initial portion of a CMR scan is dedicated to structural imaging, including cardiac size, wall thickness, morphology and function.^[9,10]^ This involves a combination of cine images to capture motion of both the right and left ventricles, and measurements (*e.g*., left ventricular [LV] ejection fraction [LVEF]). These measurements performed using CMR are generally more reproducible and accurate than those done with TTE, allowing detection of more subtle phenotypes.^[10]^ Specific anatomical regions may be better visualised with CMR than TTE, including the LV apex, lateral wall, basal septum and right ventricle.^[10]^ CMR can also overcome specific limitations to the quality of TTE images *e.g*., obesity or respiratory disease,^[11]^ and has higher spatial resolution and can image larger, unrestricted fields of view.

### Late Gadolinium Enhancement (LGE)

**Figure 1:**
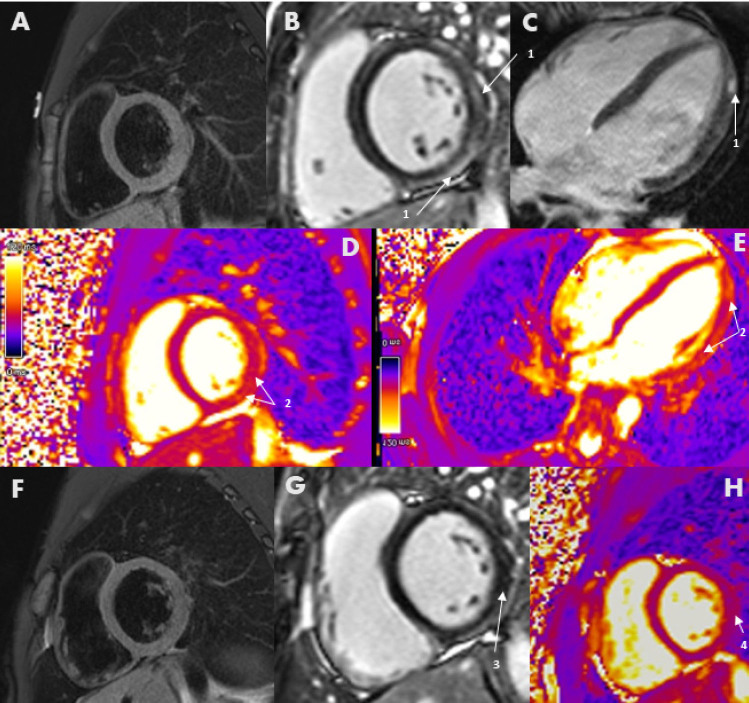
Acute Myocarditis. A Short-axis view of subtle T2 double-inversion recovery changes. B short & C long-axis view of epicardial late gadolinium enhancement (LGE) within the lateral segments of the left ventricle (1). D short & E long axis T2 colour maps confirm abnormal T2 signal within the left ventricular lateral segments (2), meeting Lake Louise criteria for myocarditis. The scale for the T2 colour maps is shown on the left, with purple representing myocardium with normal T2 relaxation times and orange representing myocardium with prolonged T2 relaxation times. F & G Follow up imaging post-resolution of myocarditis, showing mild residual LGE indicating post-inflammatory fibrosis (3). H Follow-up T2 mapping showing resolution of T2 abnormalities (4).

Post-contrast imaging utilising LGE is key in identifying myocardial fibrosis. While fibrosis can result from many pathologies, its pattern and distribution can help to identify disease aetiology, while its extent may assist in predicting heart failure and malignant arrhythmia^[10]^ (Figure 1). Gadolinium contrast is administered and accumulates in the extracellular space on delayed phase imaging, thus highlighting this space. The signal from normal myocardium is balanced by identifying and nullifying the approximate T1 value of normal tissue.^[12]^ The concentration of gadolinium becomes higher in areas with expansion of ECV, including those of necrosis and both macro- and micro-fibrosis.^[10,12]^ LGE can be reported visually or quantitively. Diffuse and/or mild delayed enhancement can be difficult to detect qualitatively. For this reason, tissue mapping is important. Different acquisition techniques and contrast agents can provide variable accuracy for LGE, although guidelines now exist for standardised protocols, reporting, interpretation and post-processing.^[8,13,14]^ The skill and experience of the radiographer and imaging physician is also crucial as undertraining and limited experience greatly affects image quality, and the sensitivity and specificity of the scan.

### Tissue Mapping & Characterisation

Mapping and quantitative analysis are more recent techniques that improve sensitivity and specificity, especially in cases of diffuse homogenous changes or with sub-clinical involvement that can be difficult to otherwise detect. T1, T2 and T2* are fundamental tissue magnetic relaxation times; measurement of these properties can be used to create a quantitative map of these values.^[10,15]^ Accurate measurements are however difficult to obtain due to artefact when the patient has implanted devices, arrhythmia or very thin myocardium.

#### T1 Mapping & ECV

In T1 mapping, a map of the measured T1 relaxation times of each pixel of myocardium is created.^[10]^ Native (pre-contrast) T1 relaxation times depend on the exact magnet and sequence used, cardiac phase and region of measurement.^[15,16]^ Accordingly, there are both measurement and biological factors implicated in variation in T1 values, which are specific to the local set-up including the exact magnet used in its local environment.^[15]^

Native T1 values are a composite signal of both the intracellular and extracellular space, and therefore can reflect abnormalities of both regions.^[15]^ Helpfully, native T1 mapping can be performed without contrast if gadolinium is contraindicated. High native T1 values can occur due to oedema (*e.g*., inflammation or infarction), increased interstitial/extracellular space (*e.g*., fibrosis), increased intracellular space (*e.g*., hypertrophy).^[10,15]^ They also overlap with normal myocardium. Reduced T1 values can occur due to lipid overload (*e.g*., lipomatous metaplasia or glycophosphingolipid accumulation) or iron overload.^[15]^

**Figure 2:**
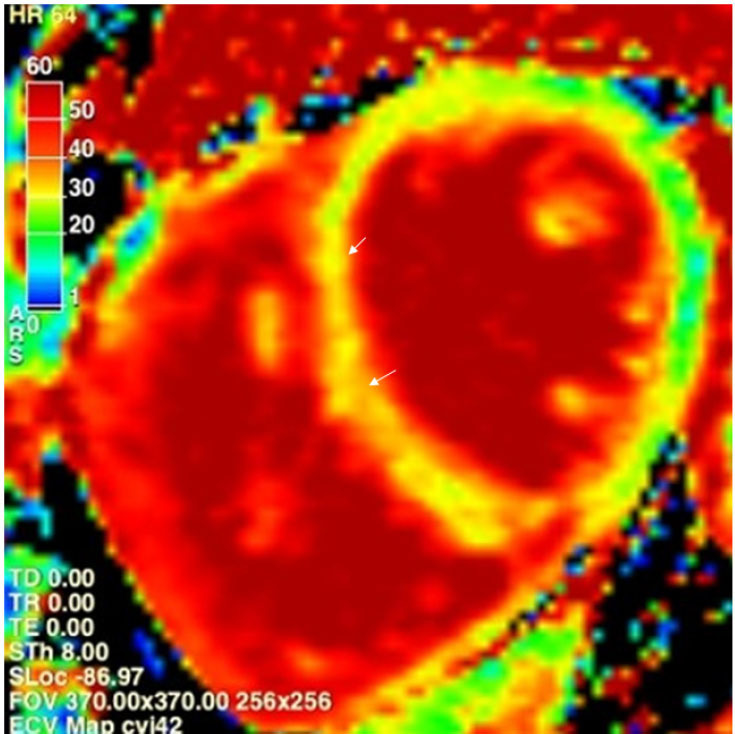
Myocardial Fibrosis. Extracellular volume (ECV) colour map showing diffuse interstitial fibrosis, more pronounced within the septum. The left-hand scale shows that a normal ECV of 25% should be in the green range. Yellow and orange myocardium indicates abnormal ECV (arrows).

Postcontrast T1 mapping with extracellular volume calculations reflects the size and composition of the extracellular space alone.^[12,15]^ Pre- and post-contrast T1 mapping can be used to calculate and map the distribution of the myocardial extracellular volume (ECV) fraction (Figure 2). This represents the proportion of the myocardium that is extracellular, as multiple pathological processes can increase the extracellular space (*e.g*., fibrosis, amyloid or oedema).^[12]^ This provides a more sensitive and specific measure of myocardial fibrosis than precontrast T1 mapping alone and can more accurately agree with histological extent of fibrosis than postcontrast T1.^[15]^ Increased ECV can also occur in amyloidosis, oedema and infiltrates, with the extent of increase in ECV more indicative of certain pathological processes.

#### T2 Mapping

T2 Double inversion recovery (DIR) images (also called “black” or “dark” blood imaging) has historically been used to detect myocardial oedema but is also useful for detection of haemorrhage and fatty infiltration. This technique can yield a false negative result for oedema detection however, as it is prone to artefact and has low sensitivity when the changes are diffuse. T2 mapping has therefore been developed to create a map of myocardial T2 relaxation times in a similar process to T1 mapping.^[10]^ T2 signal is particularly sensitive to oedema, fat and paramagnetic substances such as iron in haemoglobin.^[10]^ Global inflammation occurring in connective tissue diseases are more readily detectable by T2 mapping than by LGE^[17]^ as delayed enhancement due to oedema can be subtle.^[10]^ T2* mapping can also be used to detect and quantify iron deposition^[18]^ and haemorrhage.^[19]^

#### Diagnosis of Myocarditis

**Figure 3:**
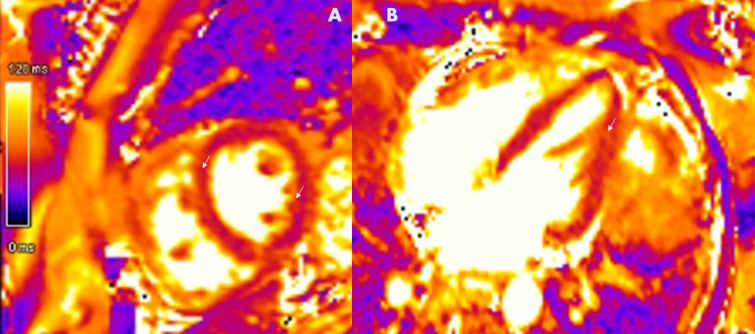
Diffuse myocardial oedema as a hallmark of connective-tissue disease associated myocarditis. A Short-axis view & B Long-axis view, T2 colour maps showing diffuse prolonged T2 relaxation times displayed as orange, confirming diffuse oedema (arrows).

Myocarditis is diagnosed according to the Lake Louise Criteria^[20]^ (Figure 1). These consensus criteria require both evidence of myocardial oedema (using a T2-based image acquisition) and a marker of associated non-ischaemic myocardial injury (abnormal native T1, ECV or LGE).^[20]^ Other supportive features include the presence of pericarditis (effusion in cine images, or abnormal LGE, T2 or T1) or systolic LV dysfunction (regional or global wall motion abnormality).^[20]^ These changes may be regional or global (Figure 3).

#### Strain Imaging

CMR assessment of myocardial strain can detect early abnormalities of myocardial deformation prior to overt myocardial dysfunction.^[21]^ Strain measures the percentage change in myocardial length as a marker of contractile function;^[21]^ this may be longitudinal, circumferential or radial, as well as global or regional.^[21]^ Measurement of longitudinal strain may identify early LV or RV dysfunction prior to deterioration of ejection fraction.^[22]^ This can be done via CMR tagging (acquiring specific sequences), or post-processing technologies applied to standard CMR techniques.^[21]^ However, different techniques may introduce variability in strain results, and further validation studies are required.^[21]^

## CMR Findings & Their Implications in SSc

The advent of CMR has identified a significantly greater burden of both myocardial fibrosis and inflammation than TTE or symptomatic assessment alone,^[6,17,23]^ in keeping with the frequency detected in historical autopsy studies.^[5]^ It must be acknowledged that as a relatively new imaging technology, our understanding of the implications of specific CMR abnormalities in SSc is evolving. However, emerging data suggest that CMR abnormalities are important prognostic indicators and potential therapeutic targets in SSc.

### Myocardial Inflammation/oedema

Chronic inflammation and myocarditis are also detected more frequently with the advent of CMR.[6,17,23,24] Left ventricular myocardial thickening diastolic dysfunction and diffuse myocardial oedema detected via T2 mapping in SSc is highly concerning for an active inflammatory myocardial lesion (*e.g*., myocarditis)^[20]^ (Figures 1, 3). This change is not identified via LGE or T1 mapping and may be missed on echocardiography. In particular, T2 mapping can help overcome the risk of false-negative results due to diffuse, subtle change not detected by readers and in those with myositis,^[24]^ because of the use of muscle signal intensity as a reference in those with skeletal myositis. This is one of the more reliable and significant markers of left heart disease in the context of SSc and provides an important opportunity for early detection and thus treatment which may prevent progression to fibrosis. In some individuals with asymptomatic early diffuse SSc (dcSSc), CMR has been able to identify T2 mapping abnormalities that progressed to florid myocarditis,^[23]^ although this is not always the case.^[6]^ Accordingly, all patients undergoing CMR with SSc should have T2 mapping performed in the short-axis view and 4-chamber planes. Resolution of T2 mapping abnormalities may also be a marker of treatment response in myocarditis (Figure 2).

### Myocardial Fibrosis

Both focal and diffuse myocardial fibrosis has been identified in asymptomatic SSc cohorts.^[17]^ T1 mapping as a marker of diffuse myocardial fibrosis in SSc can identify more abnormalities than LGE alone.^[25]^ Importantly, native T1 mapping has been proven to directly correlate with the degree of myocardial fibrosis on endomyocardial biopsy in SHI.^[24]^ Native T1 and ECV, reflecting myocardial fibrosis, have been shown to be significantly elevated in SSc compared to controls, and correlate with SSc disease activity.^[17]^ ECV quantification may also help to identify early LV disease^[26]^ (Figure 2).

#### Myocardial Fibrosis & Adverse Cardiovascular Outcomes

Recent data suggest that CMR markers of myocardial fibrosis (LGE, native T1 and ECV) are predictive of adverse cardiovascular outcomes (death, severe arrhythmia and heart failure hospitalisation) in SSc.^[27]^ In multivariable analysis, LGE appears to have a prognostic value that is incremental to elevated native T1 or ECV values, suggesting a role of both diffuse and focal myocardial fibrosis.^[27]^ In SSc patients with acute cardiac events and normal echocardiography, CMR parameters of fibrosis (LGE and T1 mapping) and oedema (T2 mapping) have been shown to predict a combined end-point of arrhythmia, angina, dyspnoea or cardiac mortality.^[28]^ Overall, these studies suggest that abnormalities on CMR indicating SHI do portend an adverse prognosis.

Data are somewhat conflicting about the role of CMR parameters in predicting the risk of arrhythmias in SSc. In SSc without overt cardiovascular disease or pulmonary arterial hypertension (PAH), no association was found between CMR-markers of focal or diffuse myocardial disease and arrhythmias.^[6]^ However, a further study showed that increased numbers of LGE-enhancing segments were associated with abnormalities on Holter monitoring in a cohort without cardiovascular risk factors but with a high proportion of PAH.^[29]^ In cohorts of those with SSc and arrhythmia, CMR parameters including LGE and T2 mapping times were shown to predict recurrence of future ventricular arrhythmia.^[30]^ It should also be noted that the burden of myocardial fibrosis in other conditions such as hypertrophic cardiomyopathy, mitral valve prolapse and arrythmogenic cardiomyopathy is a known predictor of arrhythmia risk. It is unknown whether there is a “threshold” of LGE or fibrosis that confers risk of arrhythmia/adverse outcome in SSc. Accordingly, further data are required in asymptomatic SSc cohorts to determine the predictive value of CMR for arrhythmic events, particularly in the absence of PAH. However, CMR may be a useful tool in determining likelihood of arrhythmia recurrence.

#### Myocardial Fibrosis & Impaired LV Function

CMR abnormalities may predict decline in cardiac function. Both T1 mapping and ECV have been shown to correlate with N-Terminal-pro-Brain Naturietic Peptide (NT-pro-BNP) levels.^[24]^ Subtle functional impairment may occur later in the disease course in those with early markers of fibrosis identified.^[31]^ Myocardial abnormalities may also be associated with impaired strain parameters,^[17]^ and those with myocardial fibrosis may have a lower LVEF.^[32]^ This suggests that myocardial fibrosis may have functional implications, even though relatively small regions of myocardium may be affected.^[32]^ In keeping with this idea that subtle systolic dysfunction correlates with CMR-markers of fibrosis, global longitudinal strain on echocardiography has also been shown to negatively correlate with native T1, ECV and novel cardiac biomarkers.^[33]^ This highlights the potential pathogenesis of SHI, with an early subclinical phase characterised by subtle T1/ECV changes indicative of global fibrosis causing impaired diastolic function, which may progress to more severe focal fibrosis characterised by LGE and systolic dysfunction.

### Pericardial Pathology

**Figure 4:**
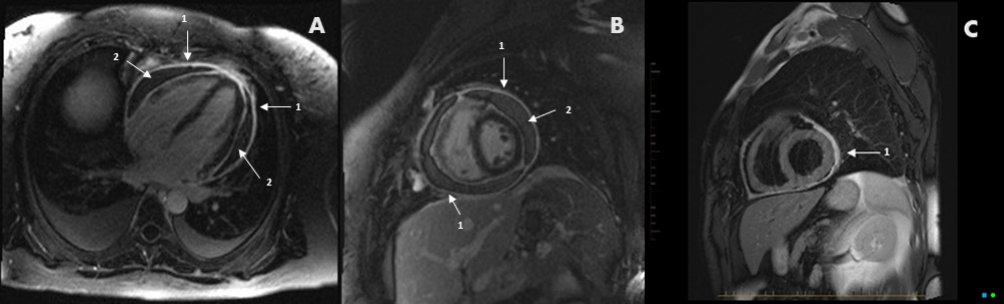
Pericarditis A long- & B short-axis views of constrictive pericarditis with overtly thickened pericardium (1) moderate volume effusion (2) and intense delayed enhancement involving both visceral and parietal pericardial layers. C Left ventricular short-axis plane T2 double inversion recovery sequence showing diffuse pericardial thickening (1) and high signal representing oedema.

CMR can provide detailed assessment of the pericardium and associated pathology, common in SSc,^[34]^ including effusion, inflammation and constrictive physiology^[35]^ (Figure 4). Pericardial thickness can be readily detected via CMR, and pericardial LGE together with T2 hyperintensity indicates active inflammation as a therapeutic target.^[35]^ Constriction physiology can be identified as abnormal ventricular interdependent on real-time cine imaging.

### Microvascular Dysfunction

Considerable interest exists as to whether microvascular dysfunction may underpin myocardial fibrosis in SHI. Historical nuclear medicine studies have identified reversible myocardial perfusion defects in those with SSc.^[36]^ Furthermore, lower LVEFs have been identified in SSc in those with greater perfusion defects on nuclear medicine imaging.^[37]^ Novel CMR techniques provide the opportunity to explore microvascular function. Dynamic CMR can assess microvascular myocardial perfusion both qualitatively and quantitatively.^[38]^ However, these are currently limited to a research setting.

### Progression over Time

Further data are required to understand the progression of CMR abnormalities over time in SSc. In one cohort (*n* = 31), CMR identified myocarditis in one participant, and new/worsening LGE in 4/31 participants with SSc without cardiovascular disease undergoing serial CMR over 33 months’ follow-up.^[39]^ All participants with new/worsening LGE or myocarditis had recent-onset dcSSc and interstitial lung disease (ILD), suggesting that while serial CMR was not particularly useful in unselected participants, it did identify new abnormalities in this high-risk group.

## CMR & Clues About the Aetiology of SHI

CMR data have provided clues about the aetiology of SHI. CMR measures of myocardial fibrosis appear more common in more severe SSc,^[17,24]^ and in dcSSc compared to limited SSc (lcSSc).^[32]^ Other data suggest that T1 mapping parameters may correlate with modified Rodnan skin score.^[24,40]^ Those with dcSSc may also have a greater extent of affected myocardium on CMR^[17]^ in keeping with findings from historic autopsy studies.^[5]^ Furthermore, myocardial fibrosis does not appear to be associated with markers of atherosclerosis.^[32]^ No difference in frequency of ischaemic heart disease or its risk factors has been noted in SSc cohorts with and without elevated T1 relaxation times.^[25]^ CMR has also helped to confirm that electrocardiographic abnormalities in SSc are more likely to be associated with SSc disease than coronary artery disease (CAD).^[41]^ Perfusion abnormalities may be more common in those with recent-onset SSc, compared to myocardial fibrosis in those with longer-standing disease,^[42]^ in keeping with the hypothesis that microvascular perfusion abnormalities lead to myocardial fibrosis in SSc.^[43]^ Serum inflammatory markers may also be associated with T1 mapping^[24]^ and CMR perfusion defects,^[32]^ supporting a potential role of biochemical inflammation in myocardial disease.

## CMR & Treatment of Myocardial Disease in SSc

No consensus guidelines exist for how CMR should be used to guide management in SSc. Given that CMR markers of myocardial oedema may be associated with tissue inflammation and myocarditis, close observation and/or commencement/intensification of disease-modifying therapy should be considered depending on the severity of these changes and associated symptoms.^[24,44]^ Management of CMR markers of myocardial fibrosis is less clear. The available data suggest that CMR markers of myocardial fibrosis are an important prognostic indicator in SSc and arguably warrant at least careful observation. The advent of antifibrotic therapies has raised the possibility of their use in myocardial fibrosis. In a small pilot study of patients treated with nintedanib for SSc-ILD, compared to a control group, myocardial ECV and RV ejection fraction were shown to stabilise in the nintedanib-treated participants and deteriorate in untreated participants.^[45]^ If changes are identified in a coronary artery distribution, then investigation and treatment of CAD should be considered. Some authors argue that the identification of diffuse myocardial fibrosis warrants consideration of vasodilatory treatment (*e.g*., calcium channel blockade), to address potential occult microvascular disease.^[46]^ Furthermore, given that CMR abnormalities are indicative of myocardial pathology, other cardioprotective medications may be beneficial, including angiotensin converting enzyme (ACE) inhibitors with a potential anti-inflammatory effect.^[47]^ This is particularly relevant in cases with borderline LV function. However, this remains unproven in SSc and accordingly should be assessed on a case-by-case basis.

## Limitations of CMR

While CMR presents a new and innovative way to characterise SHI, it has several limitations. Firstly, patient factors can limit image quality. Arrhythmia or ectopic beats can significantly degrade image quality and impinge on the accuracy of measurements. This can limit the utility of CMR in those with chronic arrhythmia or frequent ectopy. Patients are also required to perform a prolonged breath-hold to obtain diagnostic images, although modern platforms are now overcoming this with compressed sense algorithms and real time imaging. In those with significant underlying lung disease, including interstitial lung disease and PAH, as is common in SSc, patients may struggle to hold their breath long enough to obtain diagnostic images.

Additionally, CMR takes longer than average magnetic resonance imaging (MRI) scans and requires the whole head and torso of the patient to be within the magnet. Claustrophobia can thus be more problematic in patients undergoing CMR, particularly with concomitant obesity. As CMR scans take longer to perform than non-cardiac MRI, fewer patients can be imaged in the same timeframe. The level of expertise required to accurately perform and read diagnostic CMR requires sub-specialty training and experience for both radiographers and imaging physicians, meaning that fewer centres offer this service and that CMR is expensive. Access to CMR is thus limited across, and SSc may not be a funded indication for CMR in many countries and institutions. As noted above, normal values for some measurements (*e.g*., T1 values) are dependent on the local infrastructure and sequencing and are not transferrable between set-ups. Other measures may be less reproducible and therefore harder to standardise.

MRI can be contraindicated in the presence of magnetic implants or injuries. While many implanted devices are now ‘compatible’ with MRI scanners, these devices, as well as prior surgery or other intervention where metal persists in the body, may still cause artefact which precludes accurate MRI of the chest. This artefact can particularly affect tissue characterisation and mapping techniques which are essential in assessment of SHI.

Finally, gadolinium contrast is required to achieve optimal tissue characterisation. This is contraindicated in those with severe renal impairment due to the risk of nephrogenic systemic fibrosis, or a history of anaphylaxis to similar contrast agents. Repeated doses of gadolinium-based contrast agents are also not recommended in young patients, as there is potential buildup of these agents in the brain and other parts of the body. While this is less problematic with newer agents, and the clinical significance is uncertain, limiting exposure seems prudent.

## Targeting CMR Use in SSc

There is a large and under-appreciated burden of fibroinflammatory disease in SSc which is detectable via CMR. However, the implications of many findings in the absence of clinical features of heart disease are unclear. Therefore, routine use of CMR in asymptomatic cohorts is not currently recommended. In the presence of clinical indicators of cardiac disease (*e.g*., arrhythmias, chest pain, clinical cardiac failure or abnormal cardiac biomarkers), detailed structural and functional imaging and myocardial tissue characterisation is helpful in exploring a substrate for these changes. In particular, the identification of subtle myocardial oedema using T2 mapping provides an important opportunity for early disease-modifying treatment. Recognising myocardial fibrosis using T1 mapping or LGE may also help to provide important prognostic information, and with the advent of newer antifibrotic treatments, it is possible that these changes may in future be treatable. While this is unproven in SSc, it is also possible that subtle markers of myocardial dysfunction on echocardiography *e.g*., abnormal global longitudinal strain, can help to risk-stratify those who would benefit from advanced myocardial imaging.

## Future Avenues

CMR is a rapidly expanding and developing field, with an almost 6-fold increase in the number of CMR scans performed in the United Kingdom over the last decade^[48]^ and an increasing number of indications where this technique can assist with clinical decision making. Advances in software used for image acquisition and post-processing, including the advent of deep learning and artificial intelligence, provide an important opportunity for cheaper and faster scans.^[49]^ This in turn may help reduce the disparity in CMR access,^[49]^ and facilitate faster scanning.^[50]^ Artificial intelligence techniques may also help to improve reproducibility by avoiding processes that are subject to intra- and inter-observer variability.^[51]^ Furthermore, CMR could be combined with other imaging techniques *e.g*. positron emission tomography (PET) scanning. PET-MRI scanning may help provide additional and complementary information about inflammatory myocardial diseases,^[52,53]^ especially in the context of artefact, with both conventional ^18^fluorodeoxyglucose (FDG) scanning and other novel tracers.^[53]^ However, while very sensitive and specific for active oedema, this requires the addition of a considerable dose of radiation. Finally, emerging techniques may provide additional microstructural information previously only obtainable by histological investigation, including diffusion tensor imaging.^[53]^ While currently limited to research settings, these techniques would be of particular interest in SSc once more widely available.

## Conclusion

CMR noninvasively characterises the myocardium in a manner that correlates with both histopathological findings and important clinical outcomes in SSc. CMR provides insights into the pathophysiology of SHI and represents an important opportunity to further our understanding of the pathogenesis and management of SHI. While access to this imaging modality is currently limited due to the level of expertise and magnet time required to provide accurate results, recognition of its importance in SSc is key to making the test more readily available.
